# Digital Media Literacy Group for Adolescents in Child and Adolescent Psychiatry: Prospective Multicenter Single-Arm Feasibility Study

**DOI:** 10.2196/90484

**Published:** 2026-08-07

**Authors:** Ira-Katharina Petras, Sarah Wüllner, Katharin Hermenau, Kathleen Meerkamp, Michael Siniatchkin

**Affiliations:** 1 Department of Psychiatry, Psychotherapy and Psychosomatics of Children and Adolescents, RWTH Aachen University, Neuenhofer Weg 22, Aachen, North Rhine-Westphalia, 52074, Germany, 49 2418037965; 2 Institute for Interdisciplinary Conflict and Violence Research, Bielefeld University, Bielefeld, North Rhine-Westphalia, Germany; 3 Department of Psychology, Faculty of Psychology and Sports Science, Bielefeld University, Bielefeld, North Rhine-Westphalia, Germany; 4 University Clinic of Child and Adolescent Psychiatry and Psychotherapy, Evangelisches Krankenhaus Bielefeld, Bielefeld, North Rhine-Westphalia, Germany

**Keywords:** digital media literacy, adolescents, child and adolescent psychiatry, feasibility, digital mental health, mobile phone

## Abstract

**Background:**

Digital media literacy (DML) may help adolescents to handle online risks, misinformation, digital violence, and problematic media use more reflectively. However, structured interventions for adolescents in child and adolescent psychiatry (CAP), particularly in inpatient and day-treatment settings, remain scarce.

**Objective:**

This study evaluated the feasibility, acceptability, and perceived usefulness of a manualized DML group for adolescents in CAP and explored preliminary changes in digital self-efficacy and critical media literacy (CML).

**Methods:**

A prospective single-arm feasibility study with an embedded pretest-posttest and session-level process evaluation was conducted across 3 CAP clinics in Germany. The intervention comprised 6 weekly 60-minute, manualized modules covering data protection, social media use, and digital violence. Sessions were delivered in an open-group format by trained clinical staff. Recruitment was embedded in routine care, and clinical group participation was independent of study evaluation. Adolescents and facilitators completed paper-and-pencil questionnaires at baseline (T1), after each session, and post intervention or discharge (T2). Analyses were primarily descriptive; exploratory pre-post analyses used paired *t* tests.

**Results:**

The analytic feasibility sample comprised 121 adolescents who attended at least 1 module. T1 data were available for n=60 and T2 data for n=38. Overall, adolescents attended a median of 2 modules (IQR 1‐3; mean 2.46, SD 1.65, range 1‐6), and 45 attended at least 3 modules. Session-level ratings indicated moderate to high acceptability. Facilitator-rated manual fidelity was moderate to high. Retrospective T2 ratings indicated moderate to high helpfulness (n=38; mean 3.55, SD 1.18; median 4.0, IQR 3.0-4.0) and satisfaction (n=38; mean 3.79, SD 1.07; median 4.0, IQR 3.0-5.0), with no significant differences between completers and noncompleters for helpfulness (*U*=125.00; *P*=.60) or satisfaction (*U*=122.00; *P*=.53). Among completers (≥3 modules and T2 data; n=28), didactic method acceptability was moderate (mean 2.85, SD 0.60), and perceived learning was moderate to high (mean 3.33, SD 0.95). Exploratory pre-post analyses indicated an increase in perceived self-efficacy for recognizing misinformation (n=21; T1 mean 2.94, SD 0.59; T2 mean 3.38, SD 0.63; *t*_20_=2.58; Hedges *g*=0.56, 95% CI 0.09‐1.02; *P*=.02). Overall CML increased descriptively (*P*=.07), whereas digital media self-efficacy did not change significantly (*P*=.27).

**Conclusions:**

Our manualized DML group was feasible and acceptable in routine CAP. Open-group delivery and variable treatment stays limited intervention exposure and data completeness. Findings suggest that brief DML groups may address safety- and knowledge-oriented competencies, but controlled mixed methods studies with systematic recruitment and missing-data documentation are needed.

## Introduction

### Background

Digital media offer adolescents numerous opportunities for communication, information exchange, entertainment, and learning. While adults often distinguish between online and offline worlds, youth perceive digital spaces as seamlessly integrated into their daily lives (eg, studies by Ito et al [1] and Nesi et al [2,3]). German surveys underline high levels of digital media access among adolescents, and exposure begins even earlier: 1 in 10 children in Germany aged 2-5 years already own a smartphone [4,5].

Alongside these opportunities and high levels of access, digital media also pose risks such as digital violence, cyberbullying, cybergrooming, cybercrime, misinformation, and privacy violations [6,7]. At the same time, adolescents’ understanding of data flows, privacy risks, and platform mechanisms remains limited [7,8].

### Digital Media Literacy and Reflective Media Use

The European Commission [9] defines media literacy as the confident, critical, and responsible use of digital technologies for work, learning, and participation in society. Within this framework, analytical and reflective abilities are crucial: evaluating online information, detecting manipulation, and reflecting on one’s own usage patterns [10]. In this paper, we use the term *digital media literacy* (DML) to refer specifically to adolescents’ critical and responsible engagement with social media, apps, and other online media platforms, including the ability to access, analyze, evaluate, and reflect on media content and platform mechanisms [9,11-13].

This focus is clinically relevant because reflective and deliberate media use, including proactive data protection, critical evaluation, and self-reflection, can be distinguished from more impulsive, low-reflection patterns that may increase exposure to harmful content, cyberbullying, and problematic self-presentation [14-17].

### Clinical Relevance of DML in Child and Adolescent Psychiatry

Emerging evidence underscores the growing relevance of digital media use for adolescent mental health in both educational and clinical settings [18-20]. Intensive and nonreflective internet use is linked to adverse psychosocial and mental health outcomes, including internalizing symptoms, cybervictimization-related distress, depressive symptoms, anxiety, emotional dysregulation, and suicidality [6,21]. Adolescents with mental health conditions may also differ from their nonclinical peers in how they use and experience social media. Fassi et al [19] found that in a UK sample of more than 3000 adolescents, participants with any mental health condition reported spending more time on social media and being less satisfied with the number of online friends. Clinicians also perceive digital media–related issues, such as cybervictimization and maladaptive social comparison, as increasingly relevant in everyday psychiatric care [20]. However, much of the existing evidence on adolescents’ digital media use and mental health comes from community, school-based, or mixed samples, whereas evidence from routine clinical populations remains comparatively limited [18-20,22].

For adolescents in child and adolescent psychiatry (CAP), digital media use represents both a risk factor and a therapeutic context. This is particularly relevant because problematic social media use, high gaming intensity, cybervictimization, emotion regulation difficulties, and psychiatric symptoms often co-occur [23-25].

At the same time, psychiatric treatment settings provide a unique opportunity: structured, group-based interventions can address DML alongside core therapeutic goals. Given that the average length of stay in German CAP is approximately 5 weeks (32.9 d in 2021) [26], brief, manualized programs are particularly suitable. Manualized delivery formats are needed in CAP settings to ensure consistent implementation across sites and facilitators, which is crucial for both feasibility and participant acceptance [27,28]. Integrating DML into psychiatric care therefore responds directly to clinical needs, equipping vulnerable adolescents with protective skills for safe and reflective media use.

### DML Interventions and Research Gap

Despite increasing research on DML, there is limited evidence on structured, theory-driven interventions for adolescents in CAP. Existing intervention research has primarily been conducted outside routine psychiatric care. Related school-based media literacy and digital mental health literacy interventions provide relevant but not directly transferable evidence [29,30]. Cognitive-behavioral therapy–based group interventions, such as the PROTECT (*Professioneller Umgang mit technischen Medien* [professional use of technical media]) program, have demonstrated effectiveness in reducing symptoms of gaming disorder and internet use disorder in school-based at-risk samples [31,32], but they primarily target symptom reduction rather than broader DML and have not been evaluated as routine-care group interventions in inpatient or day-treatment CAP.

A specific knowledge gap remains regarding whether a manualized, face-to-face DML group can be implemented in inpatient and day-treatment CAP, whether it is acceptable to adolescents and facilitators, and whether brief modules can address safety-, knowledge-, and reflection-oriented digital media competencies in this population. To address this gap, we developed a manualized DML group, designed for real-world implementation in routine inpatient and day-treatment care, targeting all adolescents in CAP—not only those with manifest internet use disorders.

### Objectives

The objective of this study was to evaluate the feasibility, practicality, and acceptability of a newly developed, manualized DML group for adolescents in inpatient and day-treatment CAP. Following established definitions, we conceptualized feasibility as examining whether and how a new intervention can be implemented under real-world conditions, and whether progression to more definitive evaluation is warranted [33,34]. Accordingly, we assessed feasibility in terms of (1) implementation in routine CAP care, (2) demand and exposure (recruitment and session attendance), (3) acceptability and perceived appropriateness from the perspectives of adolescents and facilitators, and (4) basic indicators of practicality (resource and workflow fit) and safety (absence of intervention-related adverse events).

Beyond these feasibility-focused aims, we expected that adolescents would report perceived learning gains related to the program’s predefined learning objectives. Finally, we conducted exploratory pre-post analyses of digital self-efficacy and critical media literacy (CML) and hypothesized that both constructs would increase from baseline (T1) to postintervention (T2).

## Methods

### Intervention

The DML group is a manualized, 6-session program developed for use in inpatient and day-treatment CAP. It was primarily designed for adolescents aged 12-18 years. Its content and structure were informed by current conceptualizations of (digital) media literacy and digital mental health, which emphasize both knowledge about online risks and rights and the ability to reflect on one’s own media use. For program development, we defined 10 overarching learning objectives to translate these conceptual considerations into concrete module content (Table 1). Each module addressed a specific subset of these learning objectives and followed a manualized session structure including a brief warm-up, psychoeducational input, interactive exercises, facilitated group discussion, and a closing reflection. A detailed overview of the module structure, duration, core content, didactic methods, materials, and facilitator guidance is provided in Multimedia Appendix 1.

**Table 1.** LOs^a^ of the digital media literacy group.

LO	Description
LO1	Know where to seek help in case of digital risks (eg, cyberbullying, sexting, grooming, harmful content).
LO2	Understand psychological effects of social media use (eg, social comparison, self-presentation, mood).
LO3	Comprehend platform mechanisms and algorithms (eg, feeds, personalization, influence on opinions and behavior).
LO4	Improve data protection skills (eg, handling of personal data, privacy implications, basic protection steps).
LO5	Become aware of digital rights and legal aspects (eg, rights in cases of digital violence, reporting options).
LO6	Learn strategies to protect oneself from harmful online content or contacts (including bystander strategies).
LO7	Apply smartphone settings for safety and regulation (eg, privacy settings, notification control, screen time).
LO8	Critically evaluate online content (eg, misinformation, quality, relevance for oneself).
LO9	Recognize age-appropriate media use (eg, suitability of content, platforms, and practices for one’s age).
LO10	Reflect on one’s own smartphone use and foster deliberate, nonautomatic usage.

a LO: Learning Objective.

Program development followed a staged iterative process with a participatory refinement phase (refer to Multimedia Appendix 1 for more details). In a first step, the clinical and research team drafted an initial 6-module version of the program and delivered it in 2 full 6-session pilot cycles on the inpatient ward. The 2 pilot cycles were conducted with different adolescent groups. During these pilot cycles, facilitators observed that some basic technical content appeared too elementary for the adolescents. After the second pilot cycle, a structured group discussion was conducted with 5 adolescents from the second pilot group, all of whom had completed all 6 modules. The discussion focused on adolescents’ perceptions of relevance, comprehensibility, and didactic methods. The adolescents confirmed that several basic smartphone-related contents were already familiar to them and suggested a clearer thematic organization. Based on these observations and feedback, the introductory smartphone module was shortened and restructured, and the manual was reorganized into 3 thematic module pairs: apps I and II, social media I and II, and digital violence I and II.

Table 2 provides an overview of the 6 modules, their core content, and the corresponding learning objectives. Each session followed a fixed structure, including a short warm-up quiz, interactive input (eg, videos and illustrative examples), facilitated group discussion, focused exploration of the topic, and a closing reflection in which participants identified their personal takeaways and discussed which aspects were most relevant to them. Groups were conducted in an open-group format to accommodate variable inpatient stays (typical size: 6‐8 participants), and 2 trained clinical staff members cofacilitated each session. Additionally, the modules were thematically self-contained and did not explicitly build on one another; this modular design was chosen deliberately to accommodate heterogeneous lengths of stay in CAP so that adolescents could still benefit from individual sessions even if they were unable to attend the full program.

**Table 2.** Overview of the modules of the digital media literacy group, core content, and learning objectives.

Number	Module	Core content	Targeted LO^a^
1	Apps I	Importance of smartphones in adolescents’ daily lives Understanding app permissions and associated risks	LO4, LO7, and LO10
2	Apps II	Definition and sensitivity of personal data Risks of data sharing and loss of control Sexting and Cybergrooming	LO1, LO4, and LO6
3	Social media I	Impact of algorithms on opinion formation and behavior Critical reflection on platform dominance and monopolies Identifying misinformation and fake news	LO3 and LO8
4	Social media II	Digital self-presentation and role models online Impact of social media on mental health	LO2, LO9, and LO10
5	Digital violence I	Forms and dynamics of digital violence Shitstorms: constructive criticism versus destructive aggression Communication and boundary-setting in digital contexts	LO1, LO6, and LO8
6	Digital violence II	Legal aspects and rights in cases of digital violence Protective strategies and practical coping Help-seeking and support services	LO1, LO4, LO5, and LO6

a LO: learning objective. Refer to Table 1 for full descriptions of LO1-LO10.

### Facilitator Training

Facilitators from all sites completed a 3-hour online training led by 2 developers of the DML group. Materials (manual, worksheets, and videos) were provided in advance; one module was practiced in full, and the others were reviewed conceptually. The primary purpose of the training was to address any open questions about the modules and their delivery. Study procedures (consent, assessment timing, and data protection) were standardized. In addition, ongoing consultation and supervision were available from the development team throughout the implementation phase, so that facilitators could seek support if questions or challenges arose during delivery.

### Study Design and Procedure

We conducted a prospective single-arm feasibility study with an embedded pretest-posttest and session-level process evaluation across 3 German CAP clinics (Bielefeld, Schleswig, and Lüneburg) between January and August 2023. The intervention comprised six 60-minute modules implemented within routine inpatient and day-treatment care. Group format, facilitator training, and evaluation assessments were integrated into clinical workflows without additional staffing or dedicated funding.

The evaluation consisted of three measurement periods: baseline (T1, before the first session attended), postsession (after each module), and postintervention (T2, either at discharge or after completion of all modules). At T1 and T2, adolescents completed paper-pencil questionnaires either in group settings or individually on the ward, depending on clinical routines. In addition, they filled out brief postsession ratings at the end of each module. Facilitators provided corresponding ratings after each session and were additionally invited to complete a brief retrospective questionnaire at T2; however, due to the very small number of responses at T2, these data from the facilitators were not analyzed further. To ensure anonymity, participants generated individual alphanumeric codes following a standardized procedure, which enabled linkage across time points. Facilitators were instructed to support adolescents in generating these codes correctly and consistently across assessments to reduce linkage errors while preserving anonymity. Cases of accidental code duplication were excluded from analyses. On average, questionnaire completion required 15-20 minutes at T1 and T2 and approximately 5 minutes post session. During the 8-month study period, the DML group was repeatedly offered as part of routine inpatient and day-treatment care at all 3 sites.

### Ethical Considerations

The study was approved by the Ethics Committee of Bielefeld University (approval 2022‐276). Written informed consent was obtained from all participating adolescents and their legal guardians before inclusion in the study. Written informed consent was also obtained from all facilitators. Participation in the study evaluation was voluntary and independent of participation in the clinical group offer. Questionnaire data were collected without names or other direct personal identifiers. Repeated questionnaires were linked using self-generated study codes; no linkage list connecting codes to participant names was created. Consent documentation and local participation lists were stored separately within each institution and were accessible only to the local study team. These lists did not contain the questionnaire codes. Data were analyzed in deidentified form. No financial or material compensation was provided for study participation.

### Participants and Recruitment

Participants were eligible if they were 10- to 18-year-old inpatients or day-treatment patients deemed able to attend groups by their treating clinicians. Although the group was originally conceptualized for adolescents aged 12 years and older, in routine inpatient care younger patients (10‐11 y) are treated on the same wards and are already regularly exposed to smartphones, social media, and related online risks. Therefore, clinicians were allowed to include 10- and 11-year-olds when they judged them to be cognitively and emotionally able to participate. Exclusion criteria were acute suicidality, acute psychosis, or other conditions precluding safe group participation. Adolescents acutely affected by cyberbullying were allowed to skip the corresponding module while attending others.

Across all sites, the group was integrated into routine clinical care. Adolescents could join the open-group intervention whenever they were present on the ward, and the group was scheduled, including mid-cycle admissions. There was no predefined waiting period after admission; participation depended on clinical appropriateness as judged by the treatment team. To maximize reach within routine care, the intervention was delivered in a fixed weekly group slot embedded in the ward schedule. Study information and consent materials were introduced during admission—by the research team in Bielefeld and by study-trained clinical staff in Schleswig and Lüneburg.

The study used a convenience sampling approach. As this was an early feasibility study, no formal sample size calculation was conducted. The study period was chosen pragmatically to allow sufficient time for introducing the group at the participating sites and for delivering several complete 6-session cycles. The final number of cycles delivered at each site resulted from routine clinical workflows, staff availability, and the predefined implementation period.

The analytic feasibility sample comprised 121 adolescents who provided consent for the evaluation and attended at least 1 module of the DML group across the 3 CAP clinics. In addition, 7 facilitators delivered modules, with at least 2 staff present per group. Because recruitment was embedded in routine clinical care, the total number of adolescents who attended the clinical group irrespective of study participation was not systematically documented across sites. Likewise, the number of adolescents approached for study participation, the number of eligible adolescents who declined, and the number who did not respond were not consistently recorded. Therefore, overall group reach as well as recruitment and refusal rates could not be calculated.

### Measures

Outcomes were assessed using adolescent and facilitator questionnaires at baseline (T1), after each session, and postintervention or discharge (T2). Measures covered sociodemographic characteristics, digital media self-efficacy (DMSE), CML, session-level acceptability, facilitator-rated manual fidelity, retrospective helpfulness and satisfaction, acceptability of didactic methods, and perceived learning.

DMSE [35] was used to assess adolescents’ perceived competence in managing digital media in everyday situations. CML [36] was included to capture adolescents’ critical evaluation of online information, attitudes toward information verification, and perceived self-efficacy in recognizing misinformation, which closely matched the intervention’s learning objectives. Session-level acceptability was assessed with adapted patient version of the Group Therapy Session Questionnaire (GTS-P) and therapist version of the Group Therapy Session Questionnaire (GTS-T) [37], because these instruments capture process indicators relevant to group-based interventions. In this study, the GTS-T was completed by the group facilitators. Facilitator-rated manual fidelity was derived from adherence-focused GTS-T items [37] to assess whether the manualized intervention was delivered as intended. Study-specific T2 items were used to assess retrospective helpfulness and satisfaction, perceived helpfulness of didactic methods, and perceived learning across the predefined learning objectives. A summary of the main measures is provided in Table 3. Detailed item numbers, response formats, scoring procedures, construct coverage, internal consistencies, and study-specific items are provided in Multimedia Appendix 2.

**Table 3.** Overview of measures and assessment procedures. Didactic method acceptability and perceived learning were restricted to completers to ensure sufficient exposure to the intervention content and format.

Measure or construct	Time point and respondent	Scoring and source
Sociodemographic variables	T1^a^; adolescents; facilitators: site and gender^b^	Study-specific self-report; used for sample description.
DMSE^c^	T1, T2^d^; adolescents	Mean score of adapted 7-item DMSE scale; higher scores indicate greater perceived competence in managing digital media [35,38].
CML^e^	T1, T2; adolescents	Mean total score and three subdomains: belief in the reliability of online information, attitudes toward information verification, and perceived self-efficacy for recognizing misinformation. Adapted from Khan and Idris [36], grounded in the Theory of Planned Behavior [39].
Session-level acceptability, patient perspective (GTS-P)^f^	Post-session; adolescents	Adapted GTS-P items assessing participation, comprehensibility, helpfulness, group atmosphere, satisfaction, perceived benefit, topic interest, and didactic methods [37]. Higher scores indicate more positive session evaluation.
Session-level acceptability and implementation, facilitator perspective (GTS-T)^g^	Post-session; facilitators	Adapted GTS-T items assessing facilitator-rated patient engagement, session acceptability, clinical fit, implementation, manual adherence, and facilitator satisfaction [37]. Higher scores indicate more positive facilitator-rated session process and implementation.
Manual Fidelity Index	Post-session; facilitators	Mean of three adherence-focused GTS-T items covering manual fidelity, successful implementation, and deviation from the manual. Higher scores indicate greater fidelity.
Retrospective helpfulness and satisfaction	T2; adolescents with T2 data and ≥1 attended module	Two study-specific single-item ratings; higher scores indicate greater perceived helpfulness and satisfaction.
Didactic method acceptability	T2; adolescents ≥3 attended modules + T2	Study-specific mean score across ratings of quizzes, videos, discussions, PowerPoint-based input, flyers, and closing reflections. Higher scores indicate greater perceived helpfulness.
Perceived learning objectives	T2; adolescents ≥3 attended modules + T2	Study-specific mean score across 10 items derived from the predefined learning objectives (Table 1). Higher scores indicate greater perceived learning.

a T1: baseline.

b Facilitator site and gender were documented by the research team during training and implementation planning and verified after delivery.

c DMSE: digital media self-efficacy.

d T2: post intervention or discharge.

e CML: critical media literacy.

f GTS-P: Group Therapy Session Questionnaire, patient version.

g GTS-T: Group Therapy Session Questionnaire, therapist version, completed by facilitators.

### Data Handling and Statistical Analyses

All missing values were explicitly coded and treated as user-defined missing in IBM SPSS Statistics 29. For composite scores, participants were included if at least 80% of items within a scale were completed. This predefined conservative threshold was used to reduce the risk of unstable scale scores based on only a small number of answered items while retaining partially completed questionnaires. Analyses primarily relied on descriptive statistics to summarize attendance, acceptability, satisfaction, and learning outcomes. Exploratory paired *t* tests were conducted to examine pre-post changes among pre-post completers. Before inferential analyses, the distribution of the pre-post difference scores was examined visually using histograms, Q-Q plots, and boxplots and statistically using Shapiro-Wilk tests. Because the main pre-post outcomes were composite scores, means and SDs were used as the primary descriptive statistics. For single-item ordinal T2 outcomes, medians and IQRs were additionally reported for descriptive interpretation. For outcomes showing deviations from normality, nonparametric sensitivity analyses were additionally conducted: Wilcoxon signed-rank tests were used for exploratory paired pre-post comparisons, and Mann-Whitney *U* tests were used for retrospective T2 comparisons between completers and noncompleters. Moreover, 2-tailed *P* values were reported, with the significance level set at α=.05, and effect sizes were calculated using Hedges *g* due to small sample sizes.

GTS ratings were collected after each session and summarized descriptively at 2 aggregation levels. For overall item-level summaries and patient-facilitator comparisons, item scores were first averaged within adolescents and facilitator evaluation records across available sessions, and descriptive statistics were then calculated across these aggregated records. This aggregation was used to reduce the influence of participants or facilitators who contributed multiple session ratings. For module-level acceptability analyses, available session-level ratings were summarized separately for each module.

## Results

### Baseline Characteristics and Feasibility of Delivery

**Figure 1.**
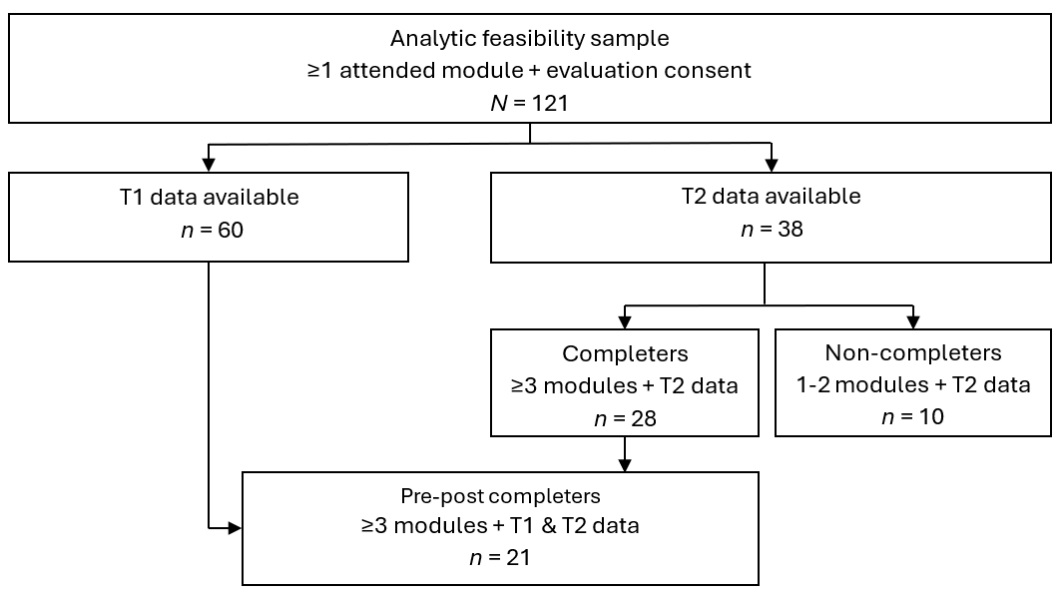
Participant flow diagram of the feasibility study. T1 and T2 questionnaire availability, completion status, and pre-post completer status are overlapping analytic subsets rather than sequential attrition categories; therefore, subgroup counts are not additive.

The analytic feasibility sample comprised 121 adolescents who provided consent for the study and attended at least 1 module. Participant flow and the overlapping analytic subsets are presented in Figure 1. Participant characteristics and intervention exposure are illustrated in Table 4. Sociodemographic data were available for a subset of participants with T1 questionnaire data. Because reasons for missing questionnaire data were not systematically documented, no formal conclusions can be drawn about the mechanisms underlying missing responses. Treatment setting and recruitment site were reported for all participants. Across sites, 7 facilitators delivered the intervention sessions—3 in Bielefeld, 2 in Schleswig, and 2 in Lüneburg. All facilitators were female. Detailed information on participant flow is presented in Figure 1.

In total, 6 full cycles of our DML group were conducted in Bielefeld, 4 in Schleswig, and 2 in Lüneburg. Adolescents attended a median of 2 (IQR 1‐3; mean 2.46, SD 1.65; range 1‐6) modules. Overall, 45 adolescents attended at least 3 modules, corresponding to at least half of the program. Among adolescents with available T2 data, completers attended a median of 5 modules (IQR 4-6), whereas noncompleters attended 1 or 2 modules (median 1.5, IQR 1‐2). In the pre-post completer subsample, adolescents attended a median of 5 modules (IQR 4-6). No adverse events related to group participation were reported.

**Table 4.** Participant characteristics of the analytic feasibility sample.

Characteristic	N	Result
Analytic feasibility sample	121	121 (100%)
Age (y)		
Mean (SD)	59	14.14 (1.90)
Range	59	10‐18
Age group		
10‐12 years	59	12 (20.3%)
13‐15 years	59	31 (52.5%)
16‐18 years	59	16 (27.1%)
Sex		
Male	56	33 (58.9%)
Female	56	16 (28.6%)
Diverse or nonbinary	56	7 (12.5%)
Site		
Bielefeld	121	46 (38.0%)
Lüneburg	121	5 (4.1%)
Schleswig	121	70 (57.9%)
Treatment context		
Inpatient ward	121	112 (92.6%)
Day-treatment clinic	121	9 (7.4%)
Modules attended		
Mean (SD)	121	2.46 (1.65)
Median (IQR)	121	2.00 (1-3)
Range	121	1‐6
Attended 1‐2 modules	121	76 (62.8%)
Attended ≥3 modules	121	45 (37.2%)

### Overall Acceptability and Satisfaction

#### Session-Level Acceptability

**Figure 2.**
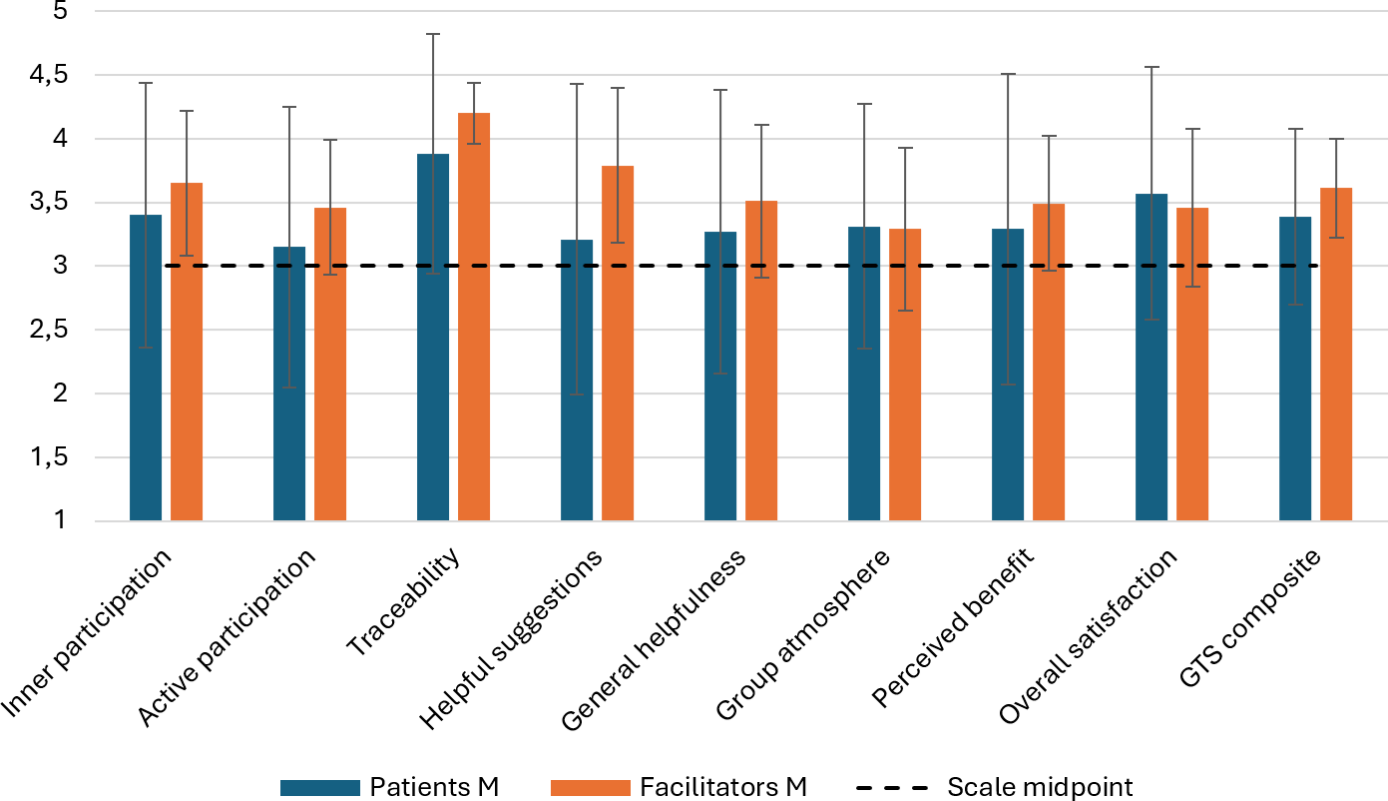
Mirrored GTS item ratings from patients and facilitators. GTS ratings were collected after each session and aggregated within adolescents or facilitator evaluation records across available sessions before descriptive summary. Patient ratings (GTS-P) are based on N=119‐121 aggregated patient-level ratings, depending on item-level missingness. Facilitator ratings (GTS-T) are based on N=18 aggregated facilitator evaluation records from n=7 facilitators. GTS composite score=mean of mirrored items. Values ≥3 indicate evaluations above neutral and reflect at least moderate endorsement. GTS: Group Therapy Session Questionnaire.

Session-level ratings indicated generally positive evaluations of the group sessions by both patients and facilitators (Figure 2). Across the 8 mirrored GTS items, mean ratings were moderate to high for both groups (mean item scores generally >3 on a 1‐5 GTS rating scale). Among adolescents “traceability” and “overall satisfaction” received the most favorable ratings, whereas “active participation” and “helpful suggestions” were rated lowest. The 2 patient-exclusive items also showed moderately positive evaluations: “interest in the session content” (mean 3.35, SD 1.15) and “helpfulness of didactic methods” (mean 3.49, SD 0.98).

In addition, facilitators’ ratings on the 5 facilitator-exclusive items were overall positive. The highest ratings were observed for “use of patient examples” (mean 4.08, SD 0.65), followed by “addressing patient concerns” (mean 3.91, SD 0.68) and “fit of the session content to patient needs” (mean 3.71, SD 0.69). “Facilitator-view group atmosphere” received the lowest rating (mean 3.32, SD 0.58), while “facilitator-specific overall satisfaction” was moderate to high (mean 3.61, SD 0.61).

#### Module-Level Acceptability

**Figure 3.**
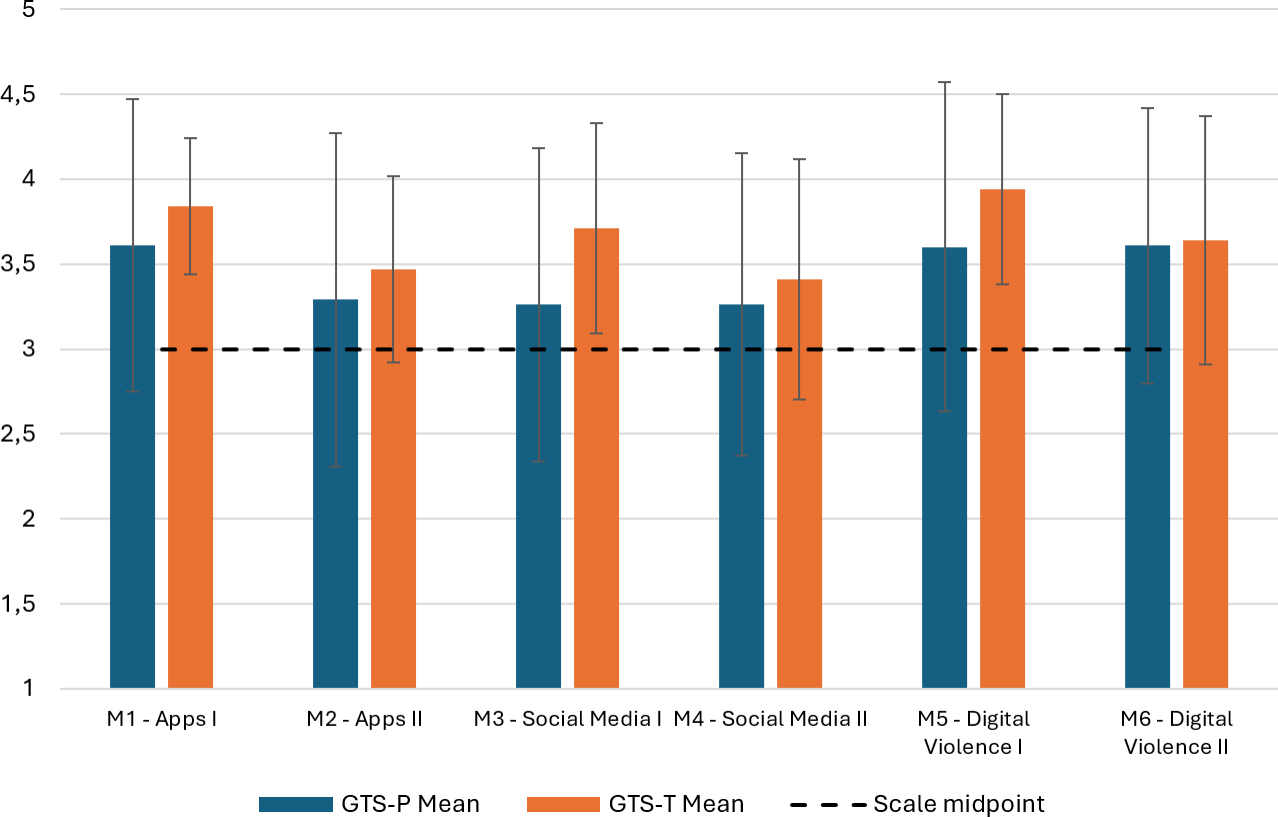
Mean session-level GTS ratings per module from patient (GTS-P) and facilitator (GTS-T) perspectives. Ratings are based on a 5-point Likert scale, with higher scores indicating greater session acceptability. Patient ratings are based on n=39‐60 available session ratings per module; facilitator ratings are based on n=9‐12 session ratings per module. Values ≥3 indicate evaluations above neutral and reflect at least moderate endorsement. GTS: Group Therapy Session Questionnaire; GTS-P: Group Therapy Session Questionnaire, patient version; GTS-T: Group Therapy Session Questionnaire, therapist version, completed by facilitator.

Module-level analyses revealed consistently positive evaluations from both patients and facilitators across all 6 modules (Figure 3). Patient ratings ranged from mean 3.26 for modules 3 (SD 0.92) and 4 (SD 0.89) to mean 3.61 (SD 0.86) for modules 1 and 6 (SD 0.81) on a 5-point Likert scale, indicating moderate to high acceptability. The highest patient ratings were observed for module 1 (apps I; n=55; mean 3.61, SD 0.86), module 5 (digital violence I; n=41; mean 3.60, SD 0.97), and module 6 (digital violence II; n=39; mean 3.61, SD 0.81). Facilitator ratings were based on 9‐12 session ratings per module and ranged from mean 3.41 (SD=0.71) to 3.94 (SD=0.56), with the highest ratings for module 1 (n=12; mean 3.84, SD 0.40) and module 5 (n=10; mean 3.94, SD 0.56). Module-specific sample sizes and descriptive statistics are reported in Multimedia Appendix 3.

#### Facilitator Manual Fidelity

Manual fidelity was generally moderate to high across modules, indicating consistent implementation of the manualized program across sites. The overall manual fidelity score, aggregated per facilitator among those who conducted at least 3 modules (n=4), was mean 3.44 (SD 0.45). Module-specific fidelity scores, based on all available facilitator ratings (n=4‐5 per module), showed highest fidelity for module 5 (digital violence I; mean 4.16, SD 0.36) and module 1 (apps I; mean 3.83, SD 0.19). Lower fidelity was observed for module 2 (apps II; mean 3.41, SD 0.85) and module 4 (social media II; mean 3.31, SD 1.12), while modules 3 and 6 demonstrated moderate fidelity (mean 3.45, SD 0.29 and mean 3.47, SD 0.78, respectively).

#### Retrospective Acceptability and Satisfaction (T2)

Among adolescents with T2 data and at least 1 attended module (n=38), retrospective ratings indicated moderate to high perceived helpfulness and satisfaction (Table 5). Ratings were comparable between completers (n=28, ≥3 modules) and noncompleters (n=10, 1‐2 modules). Because these outcomes were single-item ordinal measures and showed deviations from normality across groups, Mann-Whitney *U* tests were additionally conducted as sensitivity analyses. These showed no significant between-group differences for helpfulness (*U*=125.00, *Z*=−0.52, *P*=.60) or satisfaction (*U*=122.00, *Z*=−0.63, *P*=.53).

**Table 5.** Retrospective helpfulness and satisfaction at T2 by completion status.

Outcome	Group	n	Mean (SD)	Median (IQR)
Overall helpfulness	All T2 responders	38	3.55 (1.18)	4.0 (3.00-4.00)
Overall helpfulness	Non-completers (1‐2 modules)	10	3.40 (1.26)	3.5 (2.75-4.25)
Overall helpfulness	Completers (≥3 modules)	28	3.61 (1.17)	4.0 (3.00-4.00)
Overall satisfaction	All T2 responders	38	3.79 (1.07)	4.0 (3.00-5.00)
Overall satisfaction	Non-completers (1‐2 modules)	10	3.90 (1.20)	4.0 (3.75-5.00)
Overall satisfaction	Completers (≥3 modules)	28	3.75 (1.04)	4.0 (3.00-4.75)

#### Acceptability of Didactic Methods (T2)

Completers (n=28) evaluated 6 didactic elements on a 4-point Likert scale. Overall evaluations were moderately positive (mean 2.85, SD 0.60, 1‐4). Group discussions, videos, and quizzes were rated highest, while Microsoft PowerPoint input and flyers or brochures received lower evaluations. Closing reflections received the lowest ratings. Because these didactic elements were assessed as single-item ordinal measures, corresponding medians and interquartile ranges are additionally reported in Multimedia Appendix 4.

#### Perceived Learning (T2)

**Figure 4.**
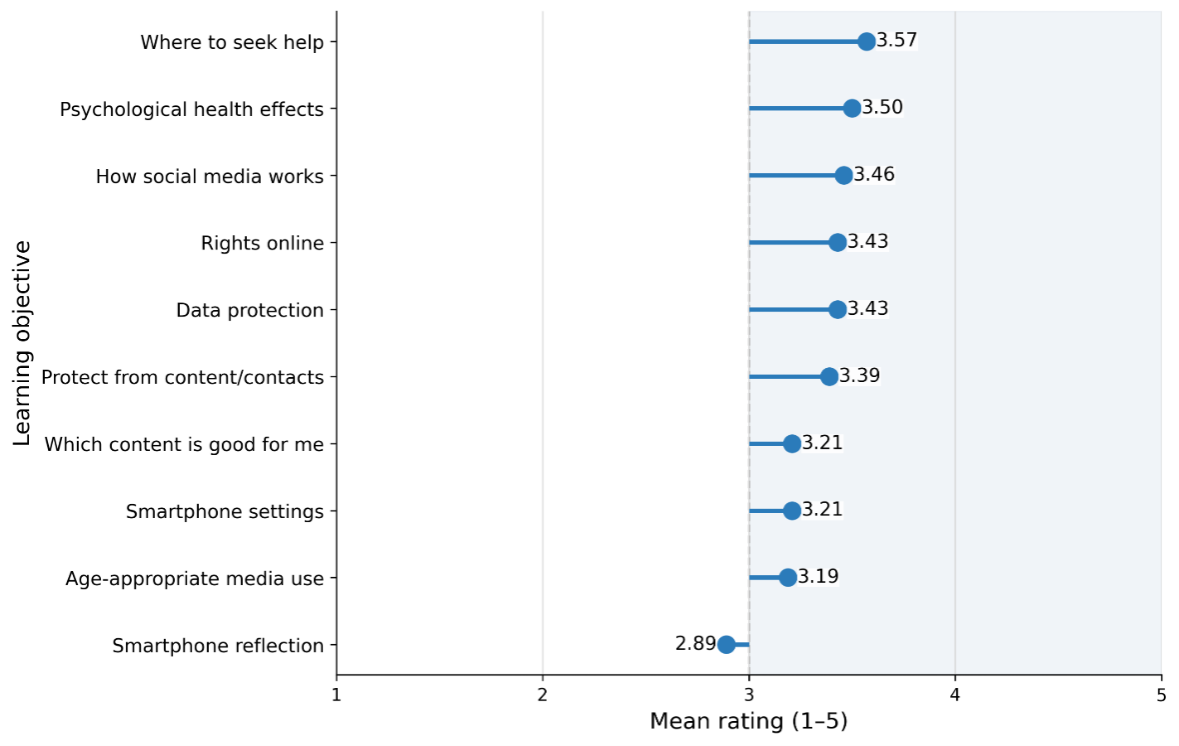
Perceived learning across ten predefined learning objectives (Completers, n=28). Ratings are based on a 5-point scale (1=strongly disagree, 5=strongly agree). The dashed vertical line marks the neutral midpoint (3). Values above 3 indicate perceived learning above the midpoint.

Among completers, the overall perceived learning composite indicated moderate to high gains (mean 3.33, SD 0.95). Figure 4 displays item-level perceived learning outcomes across the 10 predefined learning objectives. Because these learning objectives were assessed as single-item ordinal measures, between-item differences are interpreted descriptively; corresponding means, SDs, medians, and IQRs are reported in Multimedia Appendix 4. Ratings were consistently above or close to the neutral midpoint, with the highest values observed for knowing where to seek help when experiencing inappropriate online content, understanding the potential psychological effects of social media, and understanding how social media works. The lowest rating was observed for reflecting more on personal smartphone use.

#### Pre-Post Comparisons

Exploratory pre-post comparisons among pre-post completers are summarized in Table 6. CML increased descriptively but did not reach conventional statistical significance, while the perceived self-efficacy for recognizing misinformation subscale showed an exploratory increase. DMSE did not show a significant pre-post change.

**Table 6.** Exploratory pre-post comparisons among pre-post completers (n=21). Pre-post completers ≥3 modules and available T1 and T2 data. Paired-samples *t* tests are reported. Hedges g is reported as the standardized mean change, with positive values indicating improvement from T1 to T2. Normality of difference scores was inspected using histograms, Q-Q plots, boxplots, and Shapiro-Wilk tests. For outcomes with evidence of non-normality, Wilcoxon signed-rank tests were conducted as sensitivity analyses and confirmed the significant PSERM finding and the nonsignificant DMSE finding.

Outcome	T1^a^, mean (SD)	T2^b^, mean (SD)	*t* test (*df*)	Hedges *g* (95% CI)	*P* value
Critical media literacy, total	3.34 (0.61)	3.60 (0.57)	1.92 (20)	0.42 (−0.03 to 0.86)	.07^c^
PSERM^d^	2.94 (0.59)	3.38 (0.63)	2.58 (20)	0.56 (0.09 to 1.02)	.20^e^
Belief in reliability	2.89 (0.62)	2.96 (0.66)	0.63 (20)	0.14 (−0.30 to 0.57)	.54
Attitudes toward verification	3.11 (0.71)	3.17 (0.69)	0.72 (20)	0.15 (−0.28 to 0.59)	.48
DMSE^f^	3.42 (0.61)	3.55 (0.57)	1.12 (20)	0.24 (−0.19 to 0.67)	.27

a T1: baseline.

b T2: postintervention or discharge.

c *P*<.10.

d PSERM: perceived self-efficacy for recognizing misinformation.

e *P*<.05.

f DMSE: digital media self-efficacy.

## Discussion

### Principal Findings

This feasibility study examined the implementation of a manualized DML group in routine inpatient and day-treatment CAP. In line with our study objectives, we evaluated implementation in routine care, acceptability and perceived appropriateness from adolescent and facilitator perspectives, basic indicators of practicality, perceived learning gains, and exploratory pre-post changes in digital self-efficacy and CML. Overall, the program was feasible to deliver across 3 CAP sites and was accepted by adolescents and facilitators, but intervention exposure and data completeness were constrained by routine-care conditions, including open-group delivery, variable treatment stays, local scheduling procedures, and limited research resources. Perceived learning was highest for help-seeking, mental health effects of digital media, data protection, and platform mechanisms. Exploratory pre-post analyses suggested improvement in perceived self-efficacy for recognizing misinformation, whereas broader DMSE remained unchanged. Given the single-arm feasibility design and small pre-post subsample, these findings should be interpreted as preliminary feasibility signals rather than evidence of effectiveness.

### Feasibility and Acceptability in Routine CAP

Our primary feasibility objective was to determine whether our manualized DML group could be implemented under routine conditions in CAP, where digital media-related issues remain relatively new areas with limited knowledge, training, and service provision among mental health providers [20,22]. Several findings indicate that this objective was largely achieved; 121 adolescents provided evaluation consent and attended at least 1 module, all sites delivered multiple complete 6-module cycles during the 8-month study period, no adverse events were reported, and facilitators generally adhered to the manual. This suggests that a structured manual and a brief introductory training can support delivery under routine-care conditions.

At the same time, patient-level feasibility indicators highlighted important constraints. Adolescents attended a median of 2 of the 6 modules, and only 37.2% (45/121) attended at least 3 modules. At CAP in Bielefeld, local ward scheduling may have limited intervention exposure because the DML group was embedded alongside another therapeutic group offer and adolescents rotated between groups depending on their point of entry into the ward schedule. Since this site contributed a substantial proportion of cycles and participants, this procedure might have contributed to the low median number of attended modules. Therefore, limited exposure may be interpreted as a routine-care implementation constraint rather than as an indicator of low acceptability alone. This interpretation is consistent with inpatient and day-treatment CAP settings, where short and variable stays, crisis-driven discharge decisions, and tightly scheduled therapeutic programs often limit exposure to the full “intervention dose” [26,40]. Similar patterns have been reported in other inpatient and day-treatment group program evaluations, where therapeutic content may be rated positively despite incomplete session attendance [41,42]. Because reasons for nonattendance and dropout were not prospectively documented in our study, interpretations regarding attendance patterns remain tentative.

Acceptability ratings were generally positive. Adolescents and facilitators rated the sessions as understandable, relevant, and helpful, and adolescents evaluated the topics and didactic methods positively. The favorable ratings for digital-violence modules may reflect the clinical relevance of cybervictimization in psychiatric populations [20,25,43]. Variability in adolescent ratings likely reflects the heterogeneity of CAP populations in age, symptom burden, treatment phase, previous digital experiences, and baseline digital media knowledge. In this clinical context, lower active participation should not automatically be interpreted as low acceptability, because depressive withdrawal, anxiety, or reduced concentration may limit overt participation.

The positive ratings of didactic methods are consistent with evidence that young people’s engagement depends on perceived relevance, usefulness, and fit with their needs [44,45]. Together, these findings support the acceptability of addressing DML in CAP, while also underscoring the need for controlled studies under clinical real-life conditions.

### Perceived Learning and Exploratory Pre-Post Outcomes

As expected, among completers, perceived learning gains were moderate to high, and strongest for safety- and knowledge-oriented domains (knowing where to seek help, understanding psychological effects of social media, understanding platform and algorithm functioning, and data protection). These domains closely mirror competencies that are repeatedly highlighted in the literature as central for adolescents’ digital resilience and mental health [6,8,9,46,47]. Accordingly, our findings provide an important starting point for future studies that more rigorously evaluate the psychoeducational component of the DML group. Findings provide initial indications that the intervention may support specific aspects of CML: pre-post analyses showed an exploratory increase in perceived self-efficacy for recognizing misinformation with a medium standardized mean change. Overall, CML increased descriptively but did not reach conventional statistical significance. Given the small complete-case subsample and uncontrolled design, these findings should guide future trial planning rather than be interpreted as efficacy evidence [48].

The pattern of perceived learning provides useful feasibility information for refining the intervention and its evaluation. Lower ratings for more reflective objectives, such as deliberate smartphone use or age-appropriate media practices, should not only be interpreted as evidence that reflection was not stimulated by the group. Rather, they may indicate limitations in the fit between predefined learning objectives, intervention content, clinical context, and measurement approach. The group aimed to stimulate reflection through open questions, videos, facilitated discussion, and peer exchange. However, such processes may be difficult to capture with brief quantitative self-report items. In addition, smartphone use is difficult to practice behaviorally in CAP settings where smartphone access is often regulated by ward rules. Future refinements should therefore clarify reflective learning objectives, strengthen practice-based components where appropriate, and assess reflection with qualitative or mixed methods approaches.

### Clinical and Research Implications

Our findings suggest that feasibility in routine CAP depends not only on intervention content, but also on organization, staffing, workflow integration, and local ownership [27,28]. Several implementation implications can be derived from our study. First, the open-group format and modular structure of the DML group appear suitable for settings with short and variable treatment stays. Second, facilitator training should strengthen staff confidence in digital media topics, not only explain the manual. Third, the participatory development of the group content likely contributed to the positive ratings of content and methods. Co-design should therefore be considered an important design principle for future DML interventions for CAP. Fourth, future implementation requires clear local responsibilities and team-level endorsement.

Compared with related interventions, our program addresses a different implementation question. School-based media literacy programs have targeted gaming and internet use in nonclinical educational settings, whereas cognitive behavioral therapy–based programs such as PROTECT focus on adolescents at elevated risk of internet use disorder [30-32]. Digital mental health literacy interventions show promising effects on mental health literacy outcomes, particularly when psychoeducation is combined with active components, but they do not directly address DML in routine CAP [49]. The findings of this study therefore extend this literature by suggesting that a broader DML group can be delivered in inpatient and day-treatment CAP, although effectiveness remains to be tested in controlled studies. The accompanying study procedures were harder to maintain than the group delivery itself. Obtaining consent, collecting questionnaires at multiple time points, and managing follow-up around discharge required substantial staff time and affected recruitment and data completeness. This is in line with recommendations that feasibility and pilot work should explicitly address resource requirements, recruitment, and retention before proceeding to larger-scale trials [33,34]. Future studies should therefore budget dedicated resources for local coordination and routinely document attendance, nonparticipation, and drop-out reasons.

Given the heterogeneity of adolescents in CAP, future studies should examine whether DML groups need to be differentiated by age, developmental stage, baseline digital media knowledge, or online risk profile. Brief baseline screening could help clarify whether the group is best implemented as a universal routine-care offer, a developmentally tailored module, or a selective preventive intervention. Future controlled trials are needed to evaluate effectiveness under routine clinical conditions and to disentangle intervention effects from natural change. Additionally, these studies should combine low-burden session-level monitoring with structured qualitative components to better understand implementation mechanisms, subjective acceptability, and reasons for nonattendance.

### Limitations

This study has several limitations. First, recruitment and missing-data processes were incompletely documented. Because recruitment was embedded in routine care, the total number of adolescents who attended the clinical group irrespective of study participation, the number approached for evaluation participation, refusals, and nonresponses were not systematically recorded. Therefore, overall group reach, recruitment rates, refusal rates, and mechanisms of missing questionnaire data could not be determined. Site-specific scheduling procedures may also have influenced intervention exposure, but these organizational factors were not documented in sufficient detail to quantify their effect. Another limitation is that no formal qualitative postintervention data collection, such as focus groups or interviews with adolescents and facilitators, was conducted. This decision reflected the pragmatic nature of the feasibility study and the limited resources available for evaluation in routine clinical care. The study relied on brief postsession ratings and informal implementation feedback to minimize burden on patients and staff. This limited our ability to capture nuanced implementation barriers, reasons for nonattendance, and subjective acceptability. This study was a feasibility trial, and findings should be interpreted within that context only [50]. In addition, the single-arm pre-post design, small complete-case subsample, and variable intervention exposure limit statistical power and preclude causal inference. Missing baseline data may have introduced selection bias, and all outcomes relied on self-report. The study was conducted in three German CAP clinics with a small group of facilitators, all of whom identified as female, which may limit transferability to other settings and professional profiles. Potential confounding by concurrent inpatient cannot be ruled out. These limitations underline the need for controlled mixed methods evaluations before claims about effectiveness can be made.

### Conclusion

This feasibility study indicates that DML can be addressed within routine inpatient and day-treatment CAP through a brief, manualized, and modular group format. The group was perceived as relevant and acceptable and may provide a clinically useful space to discuss digital experiences that are often central to adolescents’ everyday lives but not yet systematically addressed in routine psychiatric care. DML groups may convey safety- and knowledge-oriented competencies while opening space for critical reflection and digital self-determination.

More broadly, integrating DML into CAP may help mental health services engage with adolescents’ digital realities without reducing the topic to risk or restriction alone. However, sustainable implementation depends on organizational conditions, staffing, documentation routines, and the ability to accommodate short and variable treatment stays. Future controlled mixed methods studies should evaluate effectiveness, clarify which components are useful for which subgroups, and determine how DML can be sustainably embedded into routine mental health care.

Beyond immediate clinical outcomes, embedding DML into CAP routines aligns with the European Commission’s framework of digital literacy as a prerequisite for participation in society [9]. Since participation is also a central therapeutic goal in CAP, structured DML groups may contribute not only to prevention and online safety but also to adolescents’ broader psychosocial development.

## Supplementary material

10.2196/90484 Multimedia Appendix 1 Detailed structure, delivery components, and development process of the digital media literacy group.

10.2196/90484 Multimedia Appendix 2 Detailed overview of measures, assessment procedures, scoring, reliability, and study-specific items.

10.2196/90484 Multimedia Appendix 3 Available session-level GTS ratings and mean module evaluations.

10.2196/90484 Multimedia Appendix 4 Supplementary descriptive results for didactic method acceptability and perceived learning outcomes.

## Abbreviations

CAP: child and adolescent psychiatry
CML: critical media literacy
DML: digital media literacy
DMSE: digital media self-efficacy
GTS-P: patient version of the Group Therapy Session Questionnaire
GTS-T: therapist version of the Group Therapy Session Questionnaire
PROTECT: Professioneller Umgang mit technischen Medien
