# Supplementary material for: Digital Media Literacy Group for Adolescents in Child and Adolescent Psychiatry: Prospective Multicenter Single-Arm Feasibility Study
Source: JMIR Form Res. 2026 Aug 7;10:e90484. doi: 10.2196/90484 (PMC13450890; doi:10.2196/90484)
Supplement: Supplementary file 1 [file formative-v10-e90484-s001.docx]

**Multimedia Appendix 1.** Detailed structure, delivery components, and development process of the digital media literacy group.

**Table A1.** Detailed structure and delivery components of the digital media literacy group.

| Module | Thematic block | Session structure and duration | Core content | Main activities and didactic methods | Materials and facilitator guidance |
| --- | --- | --- | --- | --- | --- |
| Apps I – (Un)Wanted Permissions? | Apps and smartphone use | 50 min: warm-up quiz; smartphone relevance exercise; app-permission exercise; closing reflection | Importance of smartphones in adolescents’ daily lives; app permissions; basic privacy and safety settings | “True/false” warm-up quiz; smartphone poster exercise in small groups; discussion of personally important apps; video-based input on app permissions; worksheet exercise identifying unnecessary app permissions; closing reflection | PowerPoint, true/false cards, A3 smartphone posters, app-permission worksheets, QR-code sticker. Facilitators use adolescents’ own app examples to introduce data collection and app permissions; adolescents are encouraged to check app permissions on their own phones after the session. |
| Apps II – Everything Under Control? | Apps and data protection | 50 min: warm-up quiz; video and discussion on personal data; pro/con exercise on data sharing; optional sensitive-topic discussion; closing reflection | Personal and sensitive data; data sharing and loss of control; risks of public data disclosure; optional sexting and cybergrooming | Warm-up quiz; video-based reflection; group collection of personal data examples; pro/con group exercise on data sharing; optional discussion of sexting and cybergrooming; discussion of warning signs and help-seeking; closing reflection | PowerPoint, videos, warning-sign material, flyers/checklists. Facilitators are instructed to check whether sensitive topics are clinically appropriate for the group and to inform ward staff if potentially distressing topics such as cybergrooming are discussed. |
| Social Media I – Too Influential? | Social media and platform mechanisms | 50 min: warm-up quiz; algorithm input and discussion; platform influence/data discussion; closing reflection | Algorithms and personalized feeds; influence on opinion formation and behavior; platform dominance/data collection; optional misinformation/fake-news content | Warm-up quiz; video-based explanation of algorithms; facilitated discussion on filter effects and opinion formation; video/discussion on platform data practices; optional fake-news exercise; closing reflection | PowerPoint, videos, true/false cards, optional fake-news quiz/resources, QR-code sticker. Facilitators use guiding questions to discuss adolescents’ own experiences with algorithmic recommendations and platform influence. |
| Social Media II – How Much Reality Is In It? | Social media and mental health | 50 min: warm-up quiz; digital self-presentation exercise; discussion of social media and mental health; protective-strategy discussion; closing reflection | Digital self-presentation; realism of social media content; social comparison; role models; effects of social media on mental health; protective strategies | Warm-up quiz; estimation/reflection exercise on how “real” social media content is; video-based discussion; psychoeducational input on mental health effects; group discussion on self-protection strategies; closing reflection | PowerPoint, videos, flipchart, QR-code sticker. Facilitators guide adolescents from personal observations to broader mechanisms such as social comparison, mood effects, self-worth, and deliberate use strategies. |
| Digital Violence I – Networked and Hurt? | Digital violence | 50 min: warm-up quiz; subjective harm-rating exercise; cyberbullying/digital violence input; offline-posting exercise; closing/self-care reflection | Forms and dynamics of digital violence; cyberbullying; hate speech, trolling, bashing, shitstorms; subjective perception of harm; communication and boundary-setting | Warm-up quiz; “hurtful or harmless?” rating exercise using colored smileys; discussion of forms of digital violence; video-based discussion; offline-posting exercise with positive comments; optional self-care reflection; closing reflection | PowerPoint, colored smileys, optional bag for anonymous ratings, post-its, video, QR-code sticker. Facilitators are guided to emphasize that perceived harm is subjective and to manage divergent group reactions and potentially distressing content. |
| Digital Violence II – Together Against It | Digital violence, rights, and help-seeking | 50 min: warm-up quiz; guided discussion of youth flyer on digital violence; support-service discussion; closing reflection | Concrete examples of digital violence; legal aspects and rights; reporting options; local and online support services; help-seeking | Warm-up quiz; guided discussion of flyer “Digital Worlds – Digital Media – Digital Violence”; discussion of barriers to help-seeking; presentation of support services; video-based discussion; closing reflection | PowerPoint, youth flyer on digital violence, cyberbullying help flyer, local support services, online support services, QR-code sticker. Facilitators are instructed to prepare ward staff because the module addresses sensitive topics including sexualized digital violence; adolescents may leave or seek support if distressed. |

**Table A2.** Development and refinement process of the digital media literacy group.

| Step | Aim | Sources / input | Persons involved | Procedure | Resulting output / adaptation |
| --- | --- | --- | --- | --- | --- |
| 1. Initial conceptual development | Identify clinically relevant digital media topics for adolescents in CAP | Scientific literature on digital media literacy, digital risks, problematic internet use, cyberbullying/cybergrooming, and digital mental health; clinical guidelines/recommendations; practice-oriented resources such as youth media safety materials | Research team and clinical team; one team member worked both clinically on the adolescent inpatient ward and in the research team | Team discussions focused on relevant media-related risks in adolescence, special needs of clinical adolescents, feasibility during inpatient/day-treatment stays, and suitability for group delivery | Decision to develop a brief, manualized, modular group intervention for routine CAP care |
| 2. Definition of learning objectives and drafting of the initial manual | Translate the selected topics into predefined learning objectives and a structured group program | Results of Step 1 | Clinical and research team | Selection of six thematic sessions; development of session aims, psychoeducational content, exercises, materials, and didactic methods; definition of a priori learning objectives for the group; | Initial six-module manualized program version with predefined learning objectives |
| 3. Pilot cycle 1 | Test initial feasibility and fit in routine inpatient care | Initial manual and facilitator observations | First adolescent pilot group; facilitators | Delivery of one full six-session cycle on the adolescent inpatient ward | Observation that some basic smartphone-related content appeared too elementary for many adolescents |
| 4. Pilot cycle 2 | Further test feasibility and refine impressions from cycle 1 | Initial manual and facilitator observations | Second adolescent pilot group; facilitators | Delivery of a second full six-session cycle with a different adolescent group | Confirmation of need to revise the structure and reduce overly basic technical content |
| 5. Structured adolescent feedback | Obtain adolescent feedback after full exposure to all modules | Experiences of adolescents who completed all six pilot modules | Five adolescents from the second pilot cycle; research team | Structured group discussion following the six modules of the initial program version; focus on relevance, comprehensibility, and didactic methods | Adolescents reported that several basic smartphone-related contents were already familiar and suggested organizing the program into three broader thematic blocks |
| 6. Final revision | Adapt the intervention based on pilot observations and adolescent feedback | Facilitator observations and adolescent feedback | Research team | Revision of module structure, content, and methods | Original introductory smartphone module was substantially shortened and restructured rather than removed; selected interactive elements were retained; media addiction was not retained as a separate module; reflective media use was addressed across modules; final structure organized into Apps I/II, Social Media I/II, and Digital Violence I/II |
