# Supplementary material for: Digital Media Literacy Group for Adolescents in Child and Adolescent Psychiatry: Prospective Multicenter Single-Arm Feasibility Study
Source: JMIR Form Res. 2026 Aug 7;10:e90484. doi: 10.2196/90484 (PMC13450890; doi:10.2196/90484)
Supplement: Supplementary file 2 [file formative-v10-e90484-s002.docx]

**Multimedia Appendix 2.** Detailed overview of measures, assessment procedures, scoring, reliability, and study-specific items.

**Table A1.** Overview of measures and assessment procedures.

| **Measure / construct** | **Time point** | **Respondent** | **Items** | **Response format** | **Construct coverage and scoring** | **Reliability / source** |
| --- | --- | --- | --- | --- | --- | --- |
| Sociodemographic variables | T1 | Adolescents | Adolescents: age, gender, treatment context; facilitators: site and gender^1^ | Self-report | Used for sample description. No systematic facilitator personal data were obtained due to data protection requirements. | Study-specific |
| Digital Media Self-Efficacy (DMSE) | T1, T2 | Adolescents | 7 | 5-point Likert scale: 1 = does not apply at all, 5 = fully applies | Mean score; higher scores indicate greater perceived competence in managing digital media. | Adapted from the DMSE scale [35], based on Schwarzer and Jerusalem [36]; α = .88 |
| Critical Media Literacy (CML) | T1, T2 | Adolescents | 13 | 5-point Likert scale: 1 = strongly disagree, 5 = strongly agree | Mean total score and three subdomain scores: belief in the reliability of online information (4 Items), attitudes toward information verification (4 Items), and perceived self-efficacy for recognizing misinformation (5 Items). Higher scores indicate better critical media literacy after reverse coding where applicable. | Adapted from Khan and Idris [37], grounded in the Theory of Planned Behavior [38]. The original two sharing-behavior items were excluded because they were less aligned with the intervention focus and difficult to interpret in inpatient/day-treatment CAP, where adolescents’ independent media use is often restricted. Full scale α = .72; belief α = .84; attitudes α = .85; PSERM α = .72 |
| Group Therapy Session Questionnaire, patient version (GTS-P) | Post-session | Adolescents | 10 | 5-point Likert scale: 1 = strongly disagree, 5 = strongly agree | Session-level process and acceptability ratings, including participation, comprehensibility, helpfulness, group atmosphere, satisfaction, perceived benefit, topic interest, and didactic methods. Higher scores indicate more positive session evaluation. | Adapted from Zoubek [39]; 8 adapted GTS-P items plus 2 study-specific items (see Tab. A2); α = .89-.94 across modules |
| Group Therapy Session Questionnaire, facilitator version (GTS-T) | Post-session | Facilitators | 16 | 5-point Likert scale: 1 = strongly disagree, 5 = strongly agree | Facilitator-rated session process and implementation, including patient-related process ratings, clinical fit, response to patient concerns, facilitator satisfaction, implementation, and manual adherence. Higher scores indicate more positive facilitator-rated process and implementation. | Adapted from Zoubek [39]; prior work supports GTS-P use in group and psychoeducational settings [40-41]. GTS-T validation remains limited; α = .69-.88 across modules |
| Manual Fidelity Index | Post-session | Facilitators | 3 selected GTS-T items | 5-point Likert scale: 1 = strongly disagree, 5 = strongly agree | Mean of three adherence-focused items covering manual fidelity, successful implementation, and deviation from the manual. Deviation item was reverse-scored; higher scores indicate greater fidelity. | Derived from GTS-T; α = .75. Overall analyses included facilitators with ≥3 delivered modules; module-level scores used all available ratings |
| Retrospective helpfulness | T2 | Adolescents with T2 data and ≥1 attended module^2^ | 1 | 5-point Likert scale: 1 = does not apply at all, 5 = fully applies | Single-item ordinal outcome; higher scores indicate greater perceived helpfulness. | Study-specific item (see Tab. A3); α = NA |
| Retrospective satisfaction | T2 | Adolescents with T2 data and ≥1 attended module^3^ | 1 | 5-point Likert scale: 1 = does not apply at all, 5 = fully applies | Single-item ordinal outcome assessing overall satisfaction with the digital media literacy group; higher scores indicate greater satisfaction. | Study-specific item (see Tab. A3); inter-item correlation with helpfulness *r* = .67, *p* < .001 |
| Didactic Method Acceptability | T2 | Adolescents ≥3 attended modules +T2 | 6 | 4-point Likert scale: 1 = not helpful at all, 4 = very helpful | Mean score and item-level ratings of perceived helpfulness of didactic methods, including quizzes, videos, discussions, PowerPoint-based input, flyers, and closing reflections. | Study-specific items (see Tab. A4); α = .78 |
| Perceived Learning Objectives | T2 | Adolescents ≥3 attended modules +T2 | 10 | 5-point Likert scale: 1 = strongly disagree, 5 = strongly agree | Mean score across 10 perceived learning items derived from predefined learning objectives; higher scores indicate greater perceived learning. | Study-specific items (see Tab. A5); learning objectives reported in Table 1; α = .94 |

**Note.** ^1^ Facilitator site and gender were documented by the research team during training and implementation planning and verified after delivery.

**Table A2.** Study-specific additional GTS-P items assessed after each session.

| Added GTS-P item | Response format |
| --- | --- |
| “Today’s topic interested me.” | 1 = strongly disagree to 5 = strongly agree |
| “I liked the way we worked on today’s topic, that is, the methods.” | 1 = strongly disagree to 5 = strongly agree |

**Note.** Items were administered in German; English translations are provided.

**Table A3.** Study-specific retrospective helpfulness and satisfaction items.

| **Domain** | **Original item content, translated into English** | **Response format** |
| --- | --- | --- |
| Retrospective helpfulness | “Overall, I experienced the digital media literacy group as helpful.” | 1 = does not apply at all to 5 = fully applies |
| Retrospective satisfaction | “Overall, I am satisfied with the digital media literacy group.” | 1 = does not apply at all to 5 = fully applies |

**Note.** Items were administered in German; English translations are provided.

**Table A4.** Study-specific didactic method acceptability items.

| **Item / method** | **Response format** |
| --- | --- |
| Game: “Did you know?” | 1 = not helpful at all to 4 = very helpful |
| Video examples | 1 = not helpful at all to 4 = very helpful |
| Discussion | 1 = not helpful at all to 4 = very helpful |
| Content input via PowerPoint presentation | 1 = not helpful at all to 4 = very helpful |
| Flyers | 1 = not helpful at all to 4 = very helpful |
| Closing round: “What do I take with me?” | 1 = not helpful at all to 4 = very helpful |

**Note.** Items were administered in German; English translations are provided.

**Table A5.** Study-specific perceived learning items.

| No. | Stem: “Through the digital media literacy group, I have learned…” | Response format |
| --- | --- | --- |
| 1 | “…how I can adjust or change specific functions of my smartphone, such as app permissions.” | 1 = strongly disagree to 5 = strongly agree |
| 2 | “…how I can protect my own data online.” | 1 = strongly disagree to 5 = strongly agree |
| 3 | “…what I can do to protect myself from inappropriate content or contacts online.” | 1 = strongly disagree to 5 = strongly agree |
| 4 | “…how social media work, for example algorithms.” | 1 = strongly disagree to 5 = strongly agree |
| 5 | “…what influence social media can have on my mental health.” | 1 = strongly disagree to 5 = strongly agree |
| 6 | “…to pay better attention to which online content is good for me and which is not.” | 1 = strongly disagree to 5 = strongly agree |
| 7 | “…to think more about how I use my smartphone.” | 1 = strongly disagree to 5 = strongly agree |
| 8 | “…where I can get help if I experience something inappropriate online.” | 1 = strongly disagree to 5 = strongly agree |
| 9 | “…what rights I and other people have online.” | 1 = strongly disagree to 5 = strongly agree |
| 10 | “…what age-appropriate use of digital media means.” | 1 = strongly disagree to 5 = strongly agree |

**Note.** Items were administered in German; English translations are provided.

**Overall note.** Items were administered in German; English translations are provided. Full item wording is shown only for study-specific evaluation items developed for the present feasibility study. Full item wording of copyrighted or third-party instruments is not reproduced; these instruments are described at construct level in the main manuscript and cited accordingly.
