# Supplementary material for: Digital Media Literacy Group for Adolescents in Child and Adolescent Psychiatry: Prospective Multicenter Single-Arm Feasibility Study
Source: JMIR Form Res. 2026 Aug 7;10:e90484. doi: 10.2196/90484 (PMC13450890; doi:10.2196/90484)
Supplement: Supplementary file 3 [file formative-v10-e90484-s003.docx]

**Multimedia Appendix 3.** Available session-level GTS ratings [39] and mean module evaluations.

**Table A1.** Available session-level GTS ratings and mean module evaluations.

| Module | Patient ratings, *n* | GTS-P mean (*SD*) | Facilitator ratings, *n* | GTS-T mean (*SD*) |
| --- | --- | --- | --- | --- |
| Module 1 - Apps I | 55 | 3.61 (0.86) | 12 | 3.84 (0.40) |
| Module 2 - Apps II | 49 | 3.29 (0.98) | 11 | 3.47 (0.55) |
| Module 3 - Social Media I | 60 | 3.26 (0.92) | 11 | 3.71 (0.61) |
| Module 4 - Social Media II | 53 | 3.26 (0.89) | 9 | 3.41 (0.71) |
| Module 5 - Digital Violence I | 41 | 3.60 (0.97) | 10 | 3.94 (0.56) |
| Module 6 - Digital Violence II | 39 | 3.61 (0.81) | 10 | 3.64 (0.73) |
| Total session ratings | 297 | - | 67 | - |

***Note.*** GTS-P = Group Therapy Session Questionnaire, patient version; GTS-T = Group Therapy Session Questionnaire, facilitator version; each available post-session evaluation contributes one data point per patient or facilitator. Module-level means shown in Figure 3 were calculated from these available session-level ratings. Total session ratings represent the sum of available ratings across modules and are not unique participants or facilitators.
