# Supplementary material for: Digital Media Literacy Group for Adolescents in Child and Adolescent Psychiatry: Prospective Multicenter Single-Arm Feasibility Study
Source: JMIR Form Res. 2026 Aug 7;10:e90484. doi: 10.2196/90484 (PMC13450890; doi:10.2196/90484)
Supplement: Supplementary file 4 [file formative-v10-e90484-s004.docx]

**Multimedia Appendix 4.** Supplementary descriptive results for didactic method acceptability and perceived learning outcomes.

**Table A1.** Acceptability of didactic methods among completers at T2.

| Didactic element | *n* | Mean (*SD*) | Median (*IQR*) |
| --- | --- | --- | --- |
| Group discussions | 28 | 3.11 (0.89) | 3.00 (1) |
| Videos | 28 | 3.07 (0.98) | 3.00 (2) |
| Quizzes/games | 28 | 3.00 (0.68) | 3.00 (0) |
| Microsoft PowerPoint input | 28 | 2.75 (0.80) | 3.00 (1) |
| Flyers/brochures | 28 | 2.71 (0.94) | 3.00 (1) |
| Closing reflections | 28 | 2.48 (1.05) | 3.00 (1) |
| Overall didactic methods score | 28 | 2.85 (0.60) | - |

**Note.** Didactic methods were assessed at T2 among completers using single-item 4-point Likert measures. Means and standard deviations are reported for comparability with the main text. Because item-level responses are ordinal, medians and interquartile ranges are additionally provided. The overall didactic methods score represents the mean across the six items and is therefore primarily interpreted using mean and standard deviation.

**Table A2.** Perceived learning outcomes across 10 predefined learning objectives among completers at T2.

| Learning objective | *n* | Mean (*SD*) | Median (*IQR*) |
| --- | --- | --- | --- |
| Where to seek help | 28 | 3.57 (1.29) | 4.0 (2) |
| Psychological health effects | 28 | 3.50 (1.20) | 4.0 (1) |
| How social media works | 28 | 3.46 (1.23) | 4.0 (1) |
| Data protection | 28 | 3.43 (1.07) | 3.0 (1) |
| Rights online | 28 | 3.43 (1.20) | 4.0 (2) |
| Protect from content/contacts | 28 | 3.39 (1.03) | 4.0 (1) |
| Smartphone settings | 28 | 3.21 (1.20) | 3.0 (2) |
| Which content is good for me | 28 | 3.21 (1.13) | 3.0 (1) |
| Age-appropriate media use | 27 | 3.19 (1.18) | 3.0 (1) |
| Smartphone reflection | 28 | 2.89 (1.17) | 3.0 (2) |
| Learning composite | 28 | 3.33 (0.95) | - |

**Note.** Learning objectives were assessed at T2 among completers using single-item 5-point Likert measures. Means and standard deviations are reported for comparability with the main text and Figure 4. Because item-level responses are ordinal and showed non-normal distributions, medians and interquartile ranges are additionally provided. The learning composite represents the mean across learning items and is therefore primarily interpreted using mean and standard deviation.
